# An mHealth Framework to Improve Birth Outcomes in Benue State, Nigeria: A Study Protocol

**DOI:** 10.2196/resprot.7743

**Published:** 2017-05-26

**Authors:** Echezona Edozie Ezeanolue, Semiu Olatunde Gbadamosi, John Olajide Olawepo, Juliet Iwelunmor, Daniel Sarpong, Chuka Eze, Amaka Ogidi, Dina Patel, Chima Onoka

**Affiliations:** ^1^ Global Health Initiative School of Community Health Sciences University of Nevada Las Vegas, NV United States; ^2^ Catholic Caritas Foundation of Nigeria Abuja Nigeria; ^3^ Department of Kinesiology and Community Health University of Illinois Urbana-Champaign, IL United States; ^4^ Center for Minority Health and Health Disparities Research and Education Xavier University Louisiana, LA United States; ^5^ Vitira LLC Arlington, VA United States; ^6^ University of Nigeria Nsukka Nigeria

**Keywords:** mHealth, smart card, HIV, hepatitis B, sickle cell disease, mobile health technology, Nigeria

## Abstract

**Background:**

The unprecedented coverage of mobile technology across the globe has led to an increase in the use of mobile health apps and related strategies to make health information available at the point of care. These strategies have the potential to improve birth outcomes, but are limited by the availability of Internet services, especially in resource-limited settings such as Nigeria.

**Objective:**

Our primary objective is to determine the feasibility of developing an integrated mobile health platform that is able to collect data from community-based programs, embed collected data into a smart card, and read the smart card using a mobile phone-based app without the need for Internet access. Our secondary objectives are to determine (1) the acceptability of the smart card among pregnant women and (2) the usability of the smart card by pregnant women and health facilities in rural Nigeria.

**Methods:**

We will leverage existing technology to develop a platform that integrates a database, smart card technology, and a mobile phone-based app to read the smart cards. We will recruit 300 pregnant women with one of the three conditions—HIV, hepatitis B virus infection, and sickle cell trait or disease—and four health facilities in their community. We will use Glasgow’s Reach, Effectiveness, Adoption, Implementation, and Maintenance framework as a guide to assess the implementation, acceptability, and usability of the mHealth platform.

**Results:**

We have recruited four health facilities and 300 pregnant women with at least one of the eligible conditions. Over the course of 3 months, we will complete the development of the mobile health platform and each participant will be offered a smart card; staff in each health facility will receive training on the use of the mobile health platform.

**Conclusions:**

Findings from this study could offer a new approach to making health data from pregnant women available at the point of delivery without the need for an Internet connection. This would allow clinicians to implement evidence-based interventions in real time to improve health outcomes.

**Trial Registration:**

ClinicalTrials.gov NCT03027258; https://clinicaltrials.gov/ct2/show/NCT03027258 (Archived by WebCite at http://www.webcitation.org/6owR2D0kE)

## Introduction

### Background

An estimated 10% of the 2.8 million newborns who died worldwide in 2013 within 28 days of birth were born in Nigeria [1]. Most of these deaths were from diseases and conditions that are often associated with the quality of care around the time of childbirth and are readily preventable or treatable with effective interventions [2-4]. The most recent figure for under-5 child mortality in Nigeria is 124 per 1000 live births [5]. Among preventable diseases that contribute to these deaths and related morbidities are HIV and hepatitis B virus (HBV) infection and sickle cell disease (SCD), with their occurrence facilitated by health system challenges such as delays in accessing quality care. The outcome is that, despite the availability of evidence-based interventions for prevention, HIV and HBV infections and SCD remain endemic in sub-Saharan African countries [1,2,6].

Nigeria alone accounted for an estimated 26% of the global burden of new HIV infections among children [7,8]. Nigeria is one of the 22 priority countries identified by the World Health Organization that collectively account for nearly 90% of all new HIV infections among children annually [7,9]; it is one of only four countries with an HIV testing rate less than 20% among pregnant women [7]. Early identification of HIV-infected pregnant women remains a critical component of prevention of mother-to-child transmission (PMTCT) of HIV.

HBV infections remain endemic in Nigeria with liver cancer now the most common cause of cancer deaths [10,11]. The risk of perinatal transmission is higher when a pregnant woman is coinfected with HIV and HBV [11-16]. Administration of hepatitis B vaccine shortly after birth and hepatitis B immunoglobulin when available has been shown to greatly reduce the risk of perinatal transmission of HBV [15]. However, missed opportunities for prevention continue to occur in resource-limited settings; this is due to poor quality of care for at-risk pregnant women and their newborns related to lack of point-of-care diagnosis and early intervention at the time of delivery [17].

Nigeria has the highest burden of SCD in the world with an estimated 150,000 children born annually with the disease [6,18]. Despite the availability of inexpensive interventions, such as pneumococcal vaccination and penicillin prophylaxis, an estimated 50%-80% of these children with SCD suffer early death before their fifth birthday due to infections [19]. Identification of infants with SCD through newborn screening and early implementation of these simple interventions have been shown to decrease mortality.

Nigeria has developed a policy framework for introduction of critical information and communication technology (ICT) infrastructure by 2020 to support the efforts toward universal health coverage [20]. The Nigeria Federal Ministry of Health (FMOH), which provides leadership for the government’s health agency, seeks to establish and scale up innovative point-of-care tools, including mHealth solutions to improve patient care and shared use of health information. Such integrated approaches will support service delivery to HIV-infected patients and provision of primary care through agencies of government, including the National Agency for the Control of AIDS and the National Primary Health Care Development Agency. Previous efforts of the FMOH, supported by the United Nations Foundation and other partners, led to the development and ongoing implementation of a strategic project (project No. ICT4SOML) to support the scale-up of maternal and child health services in the country in order to save one million lives of women and children [21].

To effectively improve health outcomes and to avoid significant social and economic losses, a health system has to be suited to use health technology to inform early interventions [4]. However, Nigeria’s health system faces great challenges in tackling perinatal transmission of HIV and HBV and in reducing deaths among children with SCD. Major barriers to effective intervention include lack of adequate health facilities, limited access to health care providers, long distance to health facilities, transportation, and high out-of-pocket costs for patients. Other barriers, such as low perception of personal risk, poor access to testing sites, cost, confidentiality, and HIV-related stigma, have been identified to impair HIV testing and PMTCT completion in Nigeria [22].

Our initial focus on HIV, HBV, and SCD is based on several factors: (1) they are prevalent in the communities where the Healthy Beginning Initiative (HBI) was implemented; HIV prevalence among pregnant women was 2.7%, prevalence of hepatitis B surface antigen was 5%, and sickle cell trait was 23%; (2) they were identified by pregnant women, their male partners, and community leaders as conditions important to their health; (3) community screening for these conditions were well accepted during HBI implementation; (4) the integration of both communicable and noncommunicable health problems showcase the dual problem facing sub-Saharan Africa; and (5) evidence-based guidelines exist for the management of infants with SCD and infants at risk of HIV and HBV.

### Potential Opportunities to Improve Health Outcome Using Mobile Technologies

To maximize effectiveness of lifesaving interventions for maternal, newborn, and child health, available evidence suggests that such strategies should be delivered under the principle of continuum of care for mother and child, from pregnancy through birth, the newborn period, infancy, and childhood [4,23]. Yet, in resource-constrained settings like Nigeria, there are often delays in implementing lifesaving interventions due to difficulties in accessing care and problems with the quality of care provided [22]. Information gaps on maternal and child health interventions, as well as structural barriers and behavioral limitations on the demand side, hinder access to lifesaving interventions [24]. As a result, implementation of simple, cost-effective, culturally adapted, and sustainable interventions are needed to save more children’s lives [4].

### Integration of Mobile Health Technologies and Medical Decision Making

The use of mHealth with integrated data and medical decision algorithms has the potential to spread implementation of evidence-based interventions. As a marked departure from current practice, we propose integrated community-based screening and availability of data at the point of delivery using mHealth to enhance care. Nigeria is Africa’s largest mobile market with over 114 million mobile phone users and a high penetration of Internet services through mobile networks [25]. The use of mHealth is a feasible and cost-effective intervention for improving maternal and perinatal outcomes in Nigeria. It can be used to increase access to health information and reduce turnaround times for receipt of laboratory test results.

### Preliminary Studies

The HBI is a community-driven, congregation-based intervention that provides health education and on-site integrated laboratory testing—HIV plus hepatitis B and sickle cell genotype—during church-organized baby showers. In 2014, we demonstrated that the HBI was feasible, acceptable, and effective in increasing uptake of HIV, hepatitis B, and sickle cell genotype testing among pregnant women and identification of infected/affected women in southeast Nigeria (National Institutes of Health [NIH] grant No. R01HD075050) [22,26]. We subsequently demonstrated that when maternal results are made available at the point of delivery, clinicians were more likely to initiate antiretroviral prophylaxis for the HIV-exposed infant and give the first dose of hepatitis B vaccine to an infant born to a mother with hepatitis B surface antigen within 24 hours. Clinicians were also more likely to screen infants for SCD, who were born to mothers with sickle cell trait, to allow for early institution of penicillin prophylaxis and appropriate immunization. Until recently, the use of information technology to make prenatal data available at the point of delivery has been limited to high-income countries due to poor infrastructure in developing countries. Fortunately, the unprecedented spread of mobile technology has made it possible to develop mHealth platforms that can be used to provide similar services to hard-to-reach communities in resource-limited settings [23,27]. This has led to improved quality of care and decreased rate of unnecessary testing, and has allowed for early institution of evidence-based interventions that improve birth outcome [28-31].

### Our Proposal and Objectives

We propose to develop an integrated mobile health platform that is able to collect data in the community, integrate collected data into a smart card, and read the smart card using a mobile phone-based app without the need for Internet access.

Our primary objective is to determine the feasibility of developing this integrated mobile health platform. Our secondary objectives are to (1) determine the acceptability of the smart card among pregnant women and (2) determine the usability of the smart cards by the pregnant women and health facilities.

## Methods

### Ethical Consideration

This study was approved by the Institutional Review Board of the University of Nevada, Las Vegas, and the Nigerian National Health Research Ethics Committee. This study was registered with ClinicalTrails.gov (ClinicalTrials.gov identifier NCT0302725).

### Theoretical Framework

Glasgow’s Reach, Effectiveness, Adoption, Implementation, and Maintenance (RE-AIM) framework is used as a guide to assess the implementation of the mHealth platform. The RE-AIM framework offers a comprehensive approach to considering five dimensions important for evaluating the potential public health impact of an intervention [32]. The model includes the following: (1) *Reach*, the percent and representativeness of individuals willing to participate; (2) *Effectiveness*, the impact of the intervention on targeted outcomes and quality of life; (3) *Adoption*, the percent and representativeness of settings and intervention staff that agree to deliver a program; (4) *Implementation*, the consistency and skill with which various program elements are delivered by various staff; and (5) *Maintenance*, the extent to which individual participants maintain behavior change long term and, at the setting level, the degree to which the program is sustained over time within the organizations delivering it [32]. *Reach* and *Effectiveness* will assess feasibility and acceptability, while *Adoption*, *Implementation*, and *Maintenance* will assess usability and sustainability of the mHealth platform.

### Study Settings and Participants

Our proposed study is anchored on the intervention for Sustained Testing and Retention (iSTAR) Among HIV-infected Patients, an ongoing NIH-funded study in Benue state, north-central Nigeria (NIH grant No. R01HD087994-01). The iSTAR is based on the HBI, our previous work that demonstrated that pregnant women and their male partners can be effectively screened in the community for HIV, SCD, and HBV (NIH grant No. R01HD075050) [22,26]. In 2012, Benue state was projected to have a population of 4,768,877, with 49.6% females and a fertility rate of 4.9% [33]. According to the 2013 Nigeria Demographic and Health Survey, about 70% of the female population had attained less than a secondary education with a literacy rate of 52.8%. Most (74.8%) were farmers residing in rural areas [34]. Preliminary data from our ongoing study demonstrates that in Benue state, prevalence rates of HIV, HBV, and SCD among pregnant women is 7.8%, 11.13%, and 19.1%, respectively.

### Participant Recruitment—Healthy Beginning Initiative

A detailed description of the baby shower program has been published previously [22]. The HBI was designed and initially tested in southeastern Nigeria as a sustainable, culturally adapted, community-driven program delivered by community health advisors to identify pregnant women, implement health interventions, and support linkage to health services for women and their families. Briefly, it has three main platforms: (1) prayer sessions during church services are used to identify pregnant women, allowing for multiple opportunities to offer health interventions; (2) church-organized baby showers are used to implement interventions that include health education and on-site integrated laboratory screening, including HIV testing; and (3) baby receptions held 6-8 weeks after birth following infant baptism to enhance postdelivery follow-up and increase early infant diagnosis [22,23]. The United States President's Emergency Plan for AIDS Relief (PEPFAR)-supported implementing partner in Benue state, Caritas Nigeria, has scaled up this approach in the priority areas where the proposed study will be conducted.

### Study Procedure

#### Overview

We will leverage existing technology to develop a mobile health platform that integrates a database, smart card technology, and a mobile phone-based app to make results available at the point of care without the need for Internet connection. Subsequently, we will recruit 300 women who attend church-organized baby shower programs in the four priority local government areas and who consent to participate in the study.

#### Inclusion and Exclusion Criteria

Participants are eligible if they have a positive test result for (1) HIV, (2) hepatitis B surface antigen, and/or (3) sickle cell trait or disease, as defined by heterozygosity (AS) or homozygosity (SS), respectively, for the S variant of hemoglobin β-subunit; and (4) are residing in the local government areas where the four health facilities are situated. Those who decline to have their test results uploaded on the secure Web-based database will be excluded.

Data from the ongoing HBI will be reviewed by study staff to determine eligible participants. Trained research assistants will approach each participant independently to inform them about the study. HBI participants who consent to enroll in the study will have data collected from their community screening session stored in a secure, Web-based database. The stored data will be encrypted into a Quick Response (QR) code embedded on a *smart card* with a unique identification number printed on the outside of the card. There will be no names or any other identifiers. The smart card will be offered to each participant. The research assistants will administer a questionnaire to each participant to collect information on acceptability of the smart card. Each participant will be asked to present the smart card at prenatal visits and delivery at the selected health care facility. At the health care facility, we will identify dedicated delivery room staff that will be trained to scan the smart card and read its contents using the mobile phone app without the need for Internet connection. Mobile phones will be provided to each facility to use for the duration of the study. When a participant presents at delivery without the card, information can still be obtained from the secure, Web-based database and confirmed using the participant’s name, date of birth, and mobile phone number.

### Study Outcomes and Analysis

#### Acceptability

We will assess the proportion of pregnant women who are willing to allow health care workers to retrieve information from their card.

#### Reach and Effectiveness

Reach will be calculated as the number of enrolled pregnant women relative to the number of eligible pregnant women in the study catchment area. The effectiveness will be determined by assessing the impact of the mHealth platform on key outcomes, such as screening for HIV, HBV, and sickle cell genotype and health care utilizations for follow-up visits. This will be measured by calculating the percentage of pregnant women that use the smart cards at the point of delivery, thus reducing the need for repeat testing and reducing missed opportunities for reduction of perinatal transmission of HIV and HBV and deaths among children with SCD. The reach and effectiveness of the mHealth platform will be captured through patient records and recruitment assessments.

#### Assessing Usability by Participants

We will conduct semistructured key informant interviews and focus group discussions with participants to assess perceived social support, obstacles, or barriers with using the cards at the point of delivery. The Perceptions, Enablers, and Nurturers (PEN-3) cultural model [35-37] centralizes culture in the design, implementation, and evaluation of any health intervention. This model will be used as a guide to assess how well informed participants feel with using the tools provided (ie, the smart card), whether its values are clarified, and whether they feel supported in using it as a resource in delivering their personal health information. In particular, we will examine (1) perceptions that may contribute to or hinder use of the smart cards; (2) the enablers or community/health factors, such as the role of health care workers in influencing participants’ use of the cards; and (3) the nurturers, or the role of family, social, or community networks in reinforcing the use of the smart cards. These perceptions, enablers, and nurturers will then be examined to identify whether they are positive (ie, factors that will lead participants to engage with the mHealth platform in general), existential (ie, practices that are unique and have no harmful health consequences), and/or negative (ie, factors that lead participants not to engage with the mHealth platform). The interviews and focus group discussions will be digitally recorded and transcribed verbatim. All data will be analyzed using NVivo version 11 (QSR International) and Krueger’s framework analysis approach, which provides a clear series of steps for qualitative data analysis, including familiarization, identifying a thematic framework, indexing and charting (ie, managing the data, data reduction), mapping, and interpretation [38].

#### Assessing Usability by Health Care Workers

We will assess the proportion and representativeness of health care settings and health care workers who are willing to use the mobile health platform in the study catchment area. To achieve this, we will calculate adoption rates using the proportion of interested health care settings relative to the total number eligible for participation. For implementation, we will capture the degree to which the mHealth platform is used as intended (ie, delivery fidelity) and by trained health care workers (ie, enactment fidelity). For maintenance, we will examine the extent to which the mHealth platform is sustained as part of routine practice in the health care settings (ie, institutionalization) at least 6 months postintervention, leadership support for sustainability or in the health care settings, as well as sustainability climate (ie, providers’ perceptions of the extent to which policies and practices in the health care settings support sustainment of the mobile health platform).

Following introduction and invitation to participate in this study, an expert panel of health care workers (n=8) [39] will assess the usability of the mHealth platform using a diverse *think-aloud heuristic* protocol. Think-aloud and heuristic evaluations are the most commonly used usability evaluation methods [39]. With *think aloud*, potential users are asked to complete a set of tasks with the artifact tested (ie, mHealth platform) and verbalize their thoughts as they work on the task [39,40]. It has been found to have high face validity since the data collected reflects actual use of an artifact and not the participant’s judgment about its usability [40]. With heuristic evaluation, the experts inspect a system (ie, mHealth) and evaluate its interface against a list of recognized usability principles known as heuristics [41,42]. These heuristics, which refer to common properties of usable systems, are based on Nielsen’s set of 10 usability principles: (1) use simple and natural dialogue, (2) speak the user’s language, (3) minimize memory load, (4) be consistent, (5) provide feedback, (6) provide clearly marked exits, (7) provide shortcuts, (8) provide good error messages, (9) prevent errors, and (10) provide help and documentation [39,43]. For this study, each expert will individually evaluate the mHealth platform in two steps: navigation and analysis. In the navigation step, the expert will become familiar with the structure and scope of the mHealth platform. In the analysis step, the expert will focus on the design of the platform to determine whether it complies with Nielsen's 10 general usability principles for interaction design. Following the evaluation, a consensus meeting will be held with the experts to identify usability problems and quantify the severity of the problems based on Nielsen’s severity rating scale, which takes into account the frequency (ie, Is the problem common or rare?), impact (ie, Is it difficult or easy for end users to address?), and persistence (ie, Is it a one-time problem or does it trouble end users repeatedly?) of the problem [44]. The problems and notes from the evaluations will be analyzed using standard descriptions that will compare the number of problems among experts and their severity.

#### Conducting the Concept-Mapping Sessions

We will use concept mapping to gather crucial information from stakeholders regarding sustainability of the overall mHealth platform in the study area. Concept mapping is a structured, participatory, mixed-methods approach to data collection that engages stakeholders in the research and theory generation process [45,46]. It integrates qualitative data with quantitative multivariate statistical analyses to describe ideas on a topic of interest and represent these ideas visually through a series of related two-dimensional maps [47]. It will be used in this study to assess the factors that may facilitate or limit the continued use of the mHealth platform in the study area over time. The recommended minimum number of participants for concept mapping is 40 [47]. We seek to recruit approximately 60 participants in order to enable subgroup analyses and to allow for modest attrition rates at each step of the concept-mapping exercise. Baseline demographics will be collected from all participants.

The concept-mapping sessions will be conducted in the following stages:

1. Preparation Stage: In this stage, stakeholders are identified and the focus question that will guide discussions on the sustainability of the mHealth medical decision model is developed.

2. Generation Stage: In this stage, key stakeholders (ie, midwives, nurses, obstetricians, primary care physicians, study staff, etc) will participate in focus group discussions to generate as many statements as possible in response to a focus question on sustainment of the mHealth platform.

3. Structuring Stage: In this stage, the stakeholders will be asked to sort the statements they generated into similar piles that make sense to them and provide names for the piles created. The stakeholders will then rate each statement based on perceived importance (ie, How important is this factor?) and likelihood (ie, How likely is it that the factor will be sustained over time?).

4. Representation Stage: In this stage, the concept-mapping analyses will be conducted using the Concept System Global Max software program (Concept Systems Inc). Data will be used to conduct multidimensional scaling (MDS) analysis with a two-dimensional solution. The MDS analysis is based on the measurement model that assumes that the relative similarity of objects can be represented in terms of the relative distance between pairs of points [46,47]. From these analyses, coordinate estimates and a two-dimensional *point map* of distances between the statements will be generated for each set of sorted data. The two-dimensional point map is chosen for its ease and interpretability [46]. To indicate the goodness of fit, a *stress value* of the point map will be developed to determine how well the MDS solution maps the original data. While it is not possible to conduct power analysis given the nature of the data, with concept mapping, a lower stress value indicates a better fit and reflects a stronger relationship between the optimal and actual configurations [46,47]. Furthermore, hierarchical cluster analysis will be conducted using the two-dimensional x-y coordinate data obtained from the MDS analysis as input and applying Ward’s algorithm as the basis for defining clusters. This approach forces the cluster analyses to partition the MDS configuration into nonoverlapping clusters in two-dimensional space [47]. This technique will also group the outcome statements on each map such that statements placed in the same cluster will be contiguous areas of the map [45]. The resulting output will be a *cluster map*, which will reveal how the statements generated, as represented by points, are grouped.

5. Interpretation Stage: In this stage, result interpretation is typically a real-time, participatory process where stakeholders interact with the total-group ideas generated. This stage involves gathering stakeholder participants to explain and discuss the results of the concept maps, pattern matching, and go-zones. This includes examining the point map to understand which statements are most related to each other, examining the cluster maps to determine which clusters of statements were rated most important to the focus statement, examining the pattern matching to determine key areas to target based on high ratings, and examining the go-zones to determine the area of most importance for each stakeholder group.

6. Utilization Stage: In this stage, the research team will work with the stakeholders to determine the best ways to use the maps and reports produced as part of the concept-mapping procedures. Possible use of the output from concept mapping includes creating a sustainability plan, which will serve as the basis for tracking the continued use of the mHealth platform in the study catchment area.

## Results

Regarding health facility recruitment, we reviewed data from 26 primary health facilities supported by PEPFAR funds in the seven priority local government areas (LGAs) in Benue state, Nigeria. We identified six comprehensive care and treatment (CCT) sites in five priority LGAs with the highest HIV incidence in pregnant women during this period. CCT sites are primary health care centers that offer HIV testing services, antiretroviral therapy for both adults and children, and PMTCT services. We excluded two health facilities in one LGA due to poor road accessibility to the sites. We selected the remaining four health facilities for the study: (1) Father Matthias Clinic, Naka (Gwer West LGA); (2) Nongu u Kristu ke Sudan hen Tiv Comprehensive Health Center, Garagbohol (Buruku LGA); (3) Nongu u Kristu ke Sudan hen Tiv Health Center, Uchi (Tarka LGA); and (4) Mimidoo Clinic, Gungul (Konshisha LGA).

## Discussion

The critical unanswered questions in low-income countries are how to identify pregnant women at risk of infections and diseases early and how to implement interventions to improve infants’ birth outcomes and survival. Whether the availability of maternal medical information, including laboratory data at the point of delivery, will enhance the implementation of proven, evidence-based interventions for prevention has not been well demonstrated in many resource-limited settings. Our proposed study is innovative in its approach to make prenatal results available at the point of care using an integrated, non-Internet-dependent, mobile health platform to guide management of mother-infant pairs in Nigeria. If successful, this approach could become a game changer in early identification of pregnant women with diseases of interest, implementation of intervention to improve birth outcomes, and reductions in loss to follow-up between testing and birth in the country.

Key stakeholders within the ICT and health sector have demonstrated the need to support the scale-up of maternal and child health services in Nigeria through ICT in order to save one million lives of women and children [21]. Accordingly, our proposed study seeks to develop a mobile health platform that is cost-effective, readily scalable, and can be integrated with other solutions to improve efficiency in health care service delivery in the country.

Our study is conducted in the context of three conditions: HIV, HBV, and SCD. Therefore, our findings may not be generalizable to other conditions. Similarly, our choice of a nonprobability sampling technique may limit the generalizability of our findings. Infrastructure gaps, such as unreliable power supply, may hinder effective implementation at our study sites.

## Abbreviations

CCT: comprehensive care and treatment
FMOH: Federal Ministry of Health
HBI: Healthy Beginning Initiative
HBV: hepatitis B virus
ICT: information and communication technology
iSTAR: intervention for Sustained Testing and Retention
LGA: local government area
MDS: multidimensional scaling
NIH: National Institutes of Health
PEN-3: Perceptions, Enablers, and Nurturers
PEPFAR: Presidents’ Emergency Plan for AIDS Relief
PMTCT: prevention of mother-to-child transmission
QR: Quick Response
RE-AIM: Reach, Effectiveness, Adoption, Implementation, and Maintenance
SCD: sickle cell disease

## Appendix

Multimedia Appendix 1 NIH R21 Peer-Review Summary Statement.
