# Supplementary material for: An mHealth Framework to Improve Birth Outcomes in Benue State, Nigeria: A Study Protocol
Source: JMIR Res Protoc. 2017 May 26;6(5):e100. doi: 10.2196/resprot.7743 (PMC5466701; doi:10.2196/resprot.7743)
Supplement: Multimedia Appendix 1 [file resprot_v6i5e100_app1.pdf]

SUMMARY STATEMENT
(Privileged Communication)

PROGRAM CONTACT:
Laura Povlich
301-496-1653
povlichlk@mail.nih.gov

Release Date: **06/23/2015**

Application Number: **1 R21 TW010252-01**

Principal Investigator

EZEANOLUE, ECHEZONA EDOZIE MD

Applicant Organization: UNIVERSITY OF NEVADA RENO

Review Group: **ZRG1 IMST-K (50)**
Center for Scientific Review Special Emphasis Panel
Mobile Health: Technology and Outcomes in Low and Middle Income Countries

Meeting Date: **06/11/2015**
Council: **AUG 2015**
Requested Start: **09/01/2015**

RFA/PA: **PAR14-028**
PCC: **MHEALTH**
Dual IC(s): **CA, EB, HD, MH**

Project Title: **A targeted approach to sickle cell genotype screening in Nigeria**

SRG Action: **Impact Score: 25**
Next Steps: **Visit http://grants.nih.gov/grants/next_steps.htm**
Human Subjects: 30-Human subjects involved - Certified, no SRG concerns
Animal Subjects: 10-No live vertebrate animals involved for competing appl.
Gender: **2A-Only women, scientifically acceptable**
Minority: **5A-Only foreign subjects, scientifically acceptable**
Children: **1A-Both Children and Adults, scientifically acceptable**
Clinical Research - not NIH-defined Phase III Trial

Project Year	Direct Costs Requested	Estimated Total Cost
1	125,000	161,185
2	125,000	161,185
TOTAL	250,000	322,369

ADMINISTRATIVE BUDGET NOTE: The budget shown is the requested budget and has not been adjusted to reflect any recommendations made by reviewers. If an award is planned, the costs will be calculated by Institute grants management staff based on the recommendations outlined below in the COMMITTEE BUDGET RECOMMENDATIONS section.

1R21TW010252-01 EZEANOLUE, ECHEZONA

RESUME AND SUMMARY OF DISCUSSION: This Exploratory/Developmental Research application from the University of Nevada Reno, the University of Illinois Urbana Champaign, Xavier University, and the Sunrise Foundation of Nigeria seeks to develop and test the feasibility, acceptability and usability of a web-based database and medical decision system that captures results for HIV, HBV and sickle cell genotype obtained from pregnant women, stores the data in a secure, web-based database and encrypts data on a “smart card” which is given to participants for use by clinicians. An effectiveness trial will assess the impact of the use of this system on the reduction in mortality among children with sickle cell disease and the prevention of perinatal transmission of HIV and HBV infections. The primary reviewers noted that if successful, the availability of maternal records at the point of delivery would potentially increase the proportion of pregnant women who initiate antiretroviral prophylaxis for HIV-exposed infants and thereby decrease mortality. Reviewers expressed a moderately high level of enthusiasm for this application with agreement that an outstanding investigative team was addressing a significant topic in public health. However reviewers also expressed concern that the team lacked a qualitative/social sciences expert and that the application lacked a discussion of how an intervention that was developed for church-based intervention participants will be moved into hospital settings where changes in hospital practice will be required in order to read the chip. Additionally, it was noted that the involvement of personnel in Nigeria was not defined thus weakening the development of research capacity. At the conclusion of an extended discussion, enthusiasm for the significance of the topic to be addressed, the outstanding investigative team and the innovative approach to genotype screening was only somewhat dampened by the weaknesses cited for the approach and the apparent lack of development of research capacity. As a consequence, the evaluation of the final potential overall impact of the application remained outstanding.

DESCRIPTION (provided by applicant): Nigeria is one of the 22 countries identified by the WHO to account for 90% of pregnant women living with HIV. Despite expansions of HIV prevention programs in Nigeria, only 14% of pregnant women were tested for HIV; only 27% of HIV-infected pregnant women received WHO recommended antiretroviral (ARV) therapy; only 11% of HIV-exposed infants received ARV prophylaxis for prevention of mother-to-child HIV transmission (PMTCT) and only 3.9% of exposed infants received appropriate testing within two months and an estimated 51,000 infants became infected with HIV in 2013. Risk of perinatal transmission is increased when a pregnant woman is co-infected with HIV and hepatitis B virus (HBV) infection which remain endemic in Nigeria where liver cancer is now the most common cause of cancer death. Children with sickle cell disease (SCD) are also at increased risk of HIV due to frequent blood transmission and an estimated 50-80% of these children die before their 5th birthday. Despite availability of simple inexpensive interventions such as penicillin prophylaxis, hepatitis B vaccine or antiretroviral prophylaxis, implementation remains inconsistent. In 2013, we demonstrated that the Healthy Beginning Initiative [HBI], a congregation-based intervention that uses prayer session to identify pregnant women early, baby shower to implement an integrated testing (HIV- plus hepatitis B and sickle cell genotype) and baby reception for follow up is acceptable and effective in increasing HIV testing among pregnant women. For this application, we will develop and test the feasibility, acceptability and usability of a web-based database and medical decision model that captures results for HIV, HBV and sickle cell genotype obtained during HBI participants; store data in a secure, web-based database; encrypt data on a “smart card” which is given to participants, and make these data available at the point-of- delivery using a cell-phone application to read the “smart card”. Data on the web-based database can also be accessed directly using the cell phone application. Evidence exists that when clinician have maternal records available at the point of delivery, they are more likely to initiate antiretroviral prophylaxis for HIV-exposed infants,

administer first dose of hepatitis B vaccine with 24 hours for infants born to women who have positive hepatitis B surface antigen and screen infants born to mothers with sickle cell trait to allow early identification and initiation of penicillin prophylaxis for infants who have sickle cell disease. The ultimate endpoint for the Phase III trial is reduction in mortality among children with sickle cell disease and prevention of perinatal transmission of HIV and HBV infections. This proposal is collaboration among Sunrise Foundation (local PEPFAR-supported partner in Nigeria); University of Illinois Urbana-Champaign (concept mapping, focus group and key informant interviews); Xavier University (data management and analysis) and University of Nevada, Reno (overall oversight and evaluation of program effectiveness).

PUBLIC HEALTH RELEVANCE: Integrated approaches to seek, test, treat and care for pregnant women to prevent perinatal transmission of diseases and identify infected/affected infants to allow for early intervention to reduce transmission, morbidity and death are urgently needed in resource-limited settings. We propose to develop and test the feasibility, acceptability and usability of a web-based data platform and a medical decision model that is integrated with a community-based screening program for HIV, HBV and sickle cell genotype that will store data in a secure web-based database; capture data in a chip imbedded “smart card”, and use a cell phone application to read the card to make data available at the point-of-delivery. Evidence shows that when clinician have maternal records at the point of delivery, they are more likely to initiate antiretroviral prophylaxis for the HIV-exposed infant, give the first dose of hepatitis B vaccine to an infant born to a mother who is positive for hepatitis B surface antigen within 24 hours of birth and screen infants born to mothers with sickle cell trait to identify sickle cell disease and implement intervention such as penicillin prophylaxis.

CRITIQUE 1:

Significance: 2

Investigator(s): 3

Innovation: 3

Approach: 3

Environment: 3

Overall Impact: Only 14% of pregnant women in Nigeria were tested for HIV; and only 27% of HIV-infected pregnant women received WHO recommended antiretroviral (ARV) therapy; only 11% of HIV-exposed infants received ARV prophylaxis for prevention of mother-to-child HIV transmission (PMTCT) and only 3.9% of exposed infants received appropriate testing within two months and an estimated 51,000 infants became infected with HIV in 2013. Risk of perinatal transmission is increased when a pregnant woman is co-infected with HIV and hepatitis B virus (HBV) infection which remain endemic in Nigeria where liver cancer is now the most common cause of cancer death. Children with sickle cell disease (SCD) are also at increased risk of HIV due to frequent blood transmission and an estimated 50-80% of these children die before their 5th birthday. Despite availability of simple inexpensive interventions such as penicillin prophylaxis, hepatitis B vaccine or antiretroviral prophylaxis, implementation remains inconsistent.

The investigators propose to develop and test the feasibility, acceptability and usability of a web-based database and medical decision model that captures results for HIV, HBV and sickle cell genotype obtained during HBI participants; store data in a secure, web-based database; encrypt data on a “smart card” which is given to participants, and make these data available at the point-of delivery using a cell-phone application to read the “smart card”.

Data on the web-based database would also be accessed directly using the cell phone application. The authors hypothesize that availability of maternal records at the point of delivery, will likely increase the proportion of pregnant women who initiate antiretroviral prophylaxis for HIV-exposed infants, administer first dose of hepatitis B vaccine with 24 hours for infants born to women who have positive hepatitis B surface antigen and screen infants born to mothers with sickle cell trait to allow early identification and initiation of penicillin prophylaxis for infants who have sickle cell disease and ultimately decrease mortality.

1. Significance:

Strengths

- Nigeria is one of only 4 countries with HIV testing rate less than 20% among pregnant women. Early identification of HIV-infected.
- Pregnant women remain a critical component of prevention of mother-to-child transmission of HIV (PMTCT).
- Hepatitis B virus (HBV) infections remain endemic in Nigeria with liver cancer now the most common cause of cancer death.
- Nigeria has the highest burden of sickle cell disease (SCD) in the world with an estimated 150,000.
- The use of mHealth with integrated data and medical decision algorithm has the potential to spread implementation of evidence-based interventions.
- Integrated community-based screening and availability of data at point-of-delivery using mHealth to enhance care.

Weaknesses

- None.

2. Investigator(s):

Strengths

- The PI is a mid-career investigator with sufficient clinical, research expertise and leadership experience to lead the proposed project.
- Co-investigators in US and Nigeria seemed also well chosen and qualified to implement the project.

Weaknesses

- Lack of qualitative/social sciences expert on the investigative team.

3. Innovation:

Strengths

- Integration of mHealth with a successful, congregation-based (in this case churches), integrated screening program that will make prenatal results available at point-of-delivery and provide a simplified medical decision algorithm to guide medical management is innovative
- If successful, this approach could become a game-changer in early identification of pregnant women with diseases of interest, implementation of intervention to improve birth outcome and reductions in loss to follow-up between testing and birth in resource-limited settings.

Weaknesses

- None

4. Approach:

Strengths

- Study design using mixed-methods (aim 1/2) to provide feasibility and acceptability of HealthPro (web-based database, encrypted smart-card, reach and effectiveness).
- Usability and Sustainability of the interventions are also evaluated.
- Detailed Medical Decision Algorithm.
- Detailed data collection and analysis.
- Detailed concept mapping sessions.
- Ultimate plans for a Phase-3 clinical trial.

Weaknesses

- None.

5. Environment:

Strengths

- Environment of the PI and collaborating institutions are adequate for the implementation of the proposed project.

Weaknesses

- None.

Research Capacity Building:

Strengths

- North-South collaboration and technology transfer between investigators in US and Nigeria

Weaknesses

- The contribution to the proposal development from local Nigeria not clear.

Protections for Human Subjects: Acceptable Risks and/or Adequate Protections

Data and Safety Monitoring Plan (Applicable for Clinical Trials Only):

Not Applicable (No Clinical Trials)

Inclusion of Women, Minorities and Children:

- Sex/Gender: Distribution justified scientifically
- Race/Ethnicity: Distribution justified scientifically
- Inclusion/Exclusion of Children under 21: Including ages < 21 justified scientifically

Vertebrate Animals: Not Applicable (No Vertebrate Animals)

Biohazards: Not Applicable (No Biohazards)

Budget and Period of Support: Recommend as Requested

CRITIQUE 2:

Significance: 1

Investigator(s): 1

Innovation: 3

Approach: 1

Environment: 2

Overall Impact: This study proposes to adapt HealthPro, an integrated mHealth platform to make results of prenatal screening for HIV, HBV, and sickle cell available to pregnant women to present to their provider on a card at the time of delivery to facilitate the appropriate screening and/or treatment of the newborn. The team has demonstrated that the Healthy Beginning Initiative, a community driven, and congregation-based intervention to promote screening dramatically increase prenatal screening. They now want to test the effects of making that data available at the point of care when women go to deliver. The proposal did not address the plans for building mHealth research capacity in Nigeria.

1. Significance:

Strengths

- The proposed study addresses the important problem of lack of prenatal screening for three conditions which can be addressed to decrease childhood mortality rates in Nigeria: HIV, HBV, and sickle cell anemia.
- The research team has conducted important foundational research that lays the ground work for the proposed study.

Weaknesses

- None noted.

2. Investigator(s):

Strengths

- Dr. Ezeanolue is an Associate Professor of Pediatrics and Epidemiology and Vice-Chair of Pediatrics for Research at University of Nevada School of Medicine. He is currently funded by NIH to conduct a comparative effectiveness trial of congregation and clinic based approaches to prenatal HIV screening.
- This is a well-established research team that has conducted preliminary studies together and is completing an R01 that lays the foundation for the proposed study.

Weaknesses

- None noted.

3. Innovation:

Strengths

- The team proposed the adaptation of an integrated platform to store the prenatal screening data, encrypt the data onto a “smart card” and make these data available to end users at the point-of-delivery along with a treatment algorithm to influence appropriate care for at-risk infants. The overall approach to care is somewhat innovative.
- Coupling the screening for HIV, HBV, and sickle cell is an innovative way to increase screening rates. Encrypting data on the card with only the child’s blood type is another way to decrease the stigmatism associate with HIV and HBV infections.

Weaknesses

- Encrypting data on the card is somewhat innovative.

4. Approach:

- The research strategy, theoretical framework guiding the approach, data collection and analysis were very well thought out.

Weaknesses

- None noted.

5. Environment:

Strengths

- Resources appear to be adequate.
- Established relationship with a productive track record.

Weaknesses

- Plans for building research capacity in Nigeria were not delineated.

Research Capacity Building:

Strengths

- None noted.

Weaknesses

- No plan for building research capacity.

Protections for Human Subjects: Acceptable Risks and/or Adequate Protections

Data and Safety Monitoring Plan (Applicable for Clinical Trials Only):

Not Applicable (No Clinical Trials)

Inclusion of Women, Minorities and Children:

- Sex/Gender: Distribution justified scientifically
- Race/Ethnicity: Distribution justified scientifically
- Inclusion/Exclusion of Children under 21: Excluding ages < 21 justified scientifically

Vertebrate Animals: Not Applicable (No Vertebrate Animals)

Biohazards: Not Applicable (No Biohazards)

Resource Sharing Plans: Acceptable

Budget and Period of Support: Recommend as Requested

CRITIQUE 3:

Significance: 2
Investigator(s): 2
Innovation: 2
Approach: 4
Environment: 1

Overall Impact: The proposed project will develop and test the feasibility, acceptability, and usability of a web-based database and medical decision model in Nigeria that captures results for HIV, HBV and sickle cell genotype to be used at the time of delivery to reduce negative health outcomes for the infant. Combining this limited electronic medical record system and a medical decision algorithm adds to the potential impact of the intervention. The team has conducted preliminary research with the study population and has plans to test the effect of this mHealth innovation on infant/child health outcomes. There are a number of things that need clarification in their approach but if these are corrected and clarified the project has the potential to have a substantial impact on infant health outcomes in Nigeria, although the impact on the field of mHealth is unclear.

1. Significance:

Strengths

- Nigeria has a low rate of HIV testing among pregnant women and accounts for one quarter of the global burden of new HIV infections among children.
- There are no electronic health records in use and so clinicians rarely have access to crucial information at the time of delivery.

Weaknesses

- No review of the literature on what has been done on this topic and similar mHealth apps.

- The application states that “lack of adequate health facilities, limited access to health care providers, long distance to health facilities, transportation, and high out-of-pocket costs for patients” are major key barriers to effective intervention yet their mHealth application does not affect any of these factors.

2. Investigator(s):

Strengths

- The PI has experience on this topic and in the study area in Nigeria.
- The team has conducted a series of studies in Nigeria on which the proposed project builds.
- The PI has a strong research and publication record.

Weaknesses

- Dr. Sarpong’s role in the project is not articulated in his biosketch.
- The Nigerian co-Is have primarily clinical and programmatic experience.

3. Innovation:

Strengths

- Integrating a HIV, HBV, and sickle cell screening program and making test results available at point-of-delivery with a medical decision algorithm for clinicians to improve the quality of care at delivery.

Weaknesses

- No literature review is provided which makes innovation difficult to assess. There are similar mHealth interventions/programs that that have created electronic medical records via mobile phone which is a part of what the team is proposing.

4. Approach:

Strengths

- The study team has well established working relationships with the communities and have done related preliminary work on which they are building.
- The scope of the project is feasible within the R21 mechanism and will prepare the team to test the mHealth intervention in a larger randomized trial.

Weaknesses

- What is the estimated percentage of women in this area who deliver in a health facility? This is critical to know to determine if the mHealth intervention has the potential for maximal reach and generalizability.
- What kind of staff will be collecting the initial data to create the patient’s record and how will laboratory results be input? How and when these will happen is not clear.
- What is the advantage of using the “smart cards” over merely asking women for their names, DOB, and phone number at the time of delivery? Cost of the smart cards?
- Details are not provided about the focus groups: the number of participants, selection of participants, timing, etc.

- Is the purpose of the concept mapping and "think aloud heuristic" to evaluate sustainability? Or is a think aloud heuristic used to evaluate usability? Why are only 6-8 participants involved in the think aloud heuristic while 60 will be involved in concept mapping? It is not clear why these respective methods were used for these outcomes (especially sustainability) and will they generate information that is crucial to determining the usability and sustainability of the app.
- Why will only one delivery room staff be trained to use the app at each participating facility? Why not all delivery room staff?
- No detail is provided about the assessment of effectiveness. It seems that effectiveness is determined by "uptake" of using the smart cards at delivery yet the outcomes listed (screening for HIV, HBV, and sickle cell genotype, and health care utilizations for follow up visits) are not assessed, nor are the aims to assess the uptake of the actual screening as this has been established in their prior work. Furthermore, why don't women who are part of the program but forget their smartcard at delivery (but are able to be located in the database by name) included in the assessment of acceptability? Ultimately it's the patient data collection and availability of screening results data at time of delivery that is important not whether or not they remembered to bring their smartcard.
- Very little detail is provided about how the data to assess reach and effectiveness will be collected.
- The assessment of adoption (adoption rates of facilities) is inconsistent with saying that 4 health facilities will participate. It seems that these have been predetermined.

5. Environment:

Strengths

- UNSOM shows a commitment to support the PI's work on this project by allowing him 25% protected time to work on this project.
- Sunrise Foundation has strong ties and a history of working with the study community.

Weaknesses

- No academic collaborator in Nigeria.

6. Research Capacity Building:

Strengths

- None noted.

Weaknesses

- No plan is articulated.
- The role of the Nigerian co-I's in the scientific aspects of the project is limited. Their role seems mainly to be oversight of the project implementation.

Protections for Human Subjects: Acceptable Risks and/or Adequate Protections

Data and Safety Monitoring Plan (Applicable for Clinical Trials Only):

Not Applicable (No Clinical Trials)

Inclusion of Women, Minorities and Children:

- Sex/Gender: Distribution justified scientifically
- Race/Ethnicity: Distribution justified scientifically
- Inclusion/Exclusion of Children under 21: Including ages < 21 justified scientifically
- Age ranges for inclusion were not noted in the proposal.

Vertebrate Animals: Not Applicable (No Vertebrate Animals)

Biohazards: Not Applicable (No Biohazards)

Budget and Period of Support: Recommend as Requested

- However, it is unclear if there is overlap with the ongoing R01.

CRITIQUE 4:

Significance: 3
Investigator(s): 2
Innovation: 4
Approach: 3
Environment: 2

Overall Impact:

1. Significance:

Strengths

- EHR at birth could improve child outcomes.

Weaknesses

- Intervention is developed for church-based intervention participants, but requires changes in hospital practice to read app. Seems unlikely that hospital will be willing to change for a patients from the HBI, and that the one person trained to read the app will be on duty to read the app when that person comes in. Would be good to know what percentage of births in hospitals are from HBI participants.

2. Investigator(s):

Strengths

- Investigator has a track record working in Nigeria.

Weaknesses

- None.

3. Innovation:

Strengths

- EHR can improve birth outcomes. Screening data from baby shower will be entered into EHR and then use of a decision aid to guide treatment for sickle cell anemia.
- Community based intervention to reach women outside of health system.

Weaknesses

- Unclear what the reach of HealthPro will be; Requires that the pregnant woman have a smartphone; Requires that someone at the health center is trained to read it. These requirements limit reach.
- Building this around HBI, while good for phase 3, seems less logical than building around a hospital that could offer this to all of its pregnant women who will deliver there.

4. Approach:

Strengths

- None Noted

Weaknesses

- Unclear how women will bring the smartcards to the hospital. (In the phone?)
- Unclear if use of smart card will be documented in patient record or how this will be known.
- No sample size calculations. The primary outcome is unclear. Main parts of the grant seem that n=3000 but then the human subjects section says n=368 and planned enrollment says n=350. Not clear how the sample is distributed across the 3 aims.

5. Environment:

Strengths

- None Noted.

Weaknesses

- None Noted.

Research Capacity Building:

Strengths

- None.

Weaknesses

- No training plan was provided.

Protections for Human Subjects: Acceptable Risks and/or Adequate Protections

Data and Safety Monitoring Plan (Applicable for Clinical Trials Only):

Not Applicable (No Clinical Trials)

Inclusion of Women, Minorities and Children:

- Sex/Gender: Distribution justified scientifically
- Race/Ethnicity: Distribution justified scientifically
- Inclusion/Exclusion of Children under 21: Including ages < 21 justified scientifically

Vertebrate Animals: Not Applicable (No Vertebrate Animals)

Biohazards: Not Applicable (No Biohazards)

Resource Sharing Plans: Acceptable

Budget and Period of Support: Recommend as Requested

THE FOLLOWING SECTIONS WERE PREPARED BY THE SCIENTIFIC REVIEW OFFICER TO SUMMARIZE THE OUTCOME OF DISCUSSIONS OF THE REVIEW COMMITTEE, OR REVIEWERS' WRITTEN CRITIQUES, ON THE FOLLOWING ISSUES:

PROTECTION OF HUMAN SUBJECTS (Resume): ACCEPTABLE

The four issues concerning protections of human subjects were adequately addressed and risks were noted to be minimal. Samples and data to be used will be de-identified and there were no concerns.

INCLUSION OF WOMEN PLAN (Resume): ACCEPTABLE

The application stated that only women will be recruited; this was justified and therefore noted to be scientifically acceptable.

INCLUSION OF MINORITIES PLAN (Resume): ACCEPTABLE

The application stated that only foreign subjects will be recruited; this was justified and therefore noted to be scientifically acceptable.

INCLUSION OF CHILDREN PLAN (Resume): ACCEPTABLE

The application stated that both adults and children will be recruited for this study; this was justified and therefore noted to be scientifically acceptable.

COMMITTEE BUDGET RECOMMENDATIONS: The budget was recommended as requested.

NIH has modified its policy regarding the receipt of resubmissions (amended applications). See Guide Notice NOT-OD-14-074 at http://grants.nih.gov/grants/guide/notice-files/NOT-OD-14-074.html. The impact/priority score is calculated after discussion of an application by averaging the overall scores (1-9) given by all voting reviewers on the committee and multiplying by 10. The criterion scores are submitted prior to the meeting by the individual reviewers assigned to an application, and are not discussed specifically at the review meeting or calculated into the overall impact score. Some applications also receive a percentile ranking. For details on the review process, see http://grants.nih.gov/grants/peer_review_process.htm#scoring.

MEETING ROSTER

Center for Scientific Review Special Emphasis Panel
CENTER FOR SCIENTIFIC REVIEW
Mobile Health: Technology and Outcomes in Low and Middle Income Countries
ZRG1 IMST-K (50) R
June 11, 2015 - June 12, 2015

CHAIRPERSON

LIECHTY, EDWARD A, MD
PROFESSOR
DEPARTMENT OF PEDIATRICS
INDIANA UNIVERSITY
SCHOOL OF MEDICINE
INDIANAPOLIS, IN 462025210

SPRUIJT-METZ, DONNA D, PHD
RESEARCH PROFESSOR AND DIRECTOR
USC MHEALTH COLLABORATORY
CENTER FOR ECONOMIC AND SOCIAL RESEARCH
UNIVERSITY OF SOUTHERN CALIFORNIA
LOS ANGELES, CA 90089

MEMBERS

ABDULLAH, ABU SALEH, MD, PHD
ASSOCIATE PROFESSOR
DEPARTMENT OF GENERAL INTERNAL MEDICINE
BOSTON MEDICAL CENTER
BOSTON UNIVERSITY MEDICAL CAMPUS
BOSTON, MA 02118

ABROMS, LORIEN C ABROM, SCD
ASSOCIATE PROFESSOR
MILKEN INSTITUTE SCHOOL OF PUBLIC HEALTH
DEPARTMENT OF PREVENTION AND COMMUNITY HEALTH
GEORGE WASHINGTON UNIVERSITY
WASHINGTON, DC 20037

AHAMED, SHEIKH I, PHD
PROFESSOR
DEPARTMENT OF MATHEMATICS, STATISTICS
AND COMPUTER SCIENCE
MARQUETTE UNIVERSITY
MILWAUKEE, WI 53233

ASCHE, CARL , PHD
DIRECTOR AND PROFESSOR
CENTER FOR OUTCOMES RESEARCH
UNIVERSITY OF ILLINOIS COLLEGE OF MEDICINE
PEORIA, IL 61605

BARCLAY, GILLIAN R, DDS
PUBLIC HEALTH ADVISORS
GLOBAL PARTNERSHIPS FOR DEVELOPMENT
PONTIAC, MI 48340

BAUERMEISTER, JOSE A, MPH, PHD
ASSOCIATE PROFESSOR
HEALTH BEHAVIOR AND HEALTH EDUATION
UNIVERSITY OF MICHIGAN SCHOOL OF PUBLIC HEALTH
ANN ARBOR, MI 48109

BAUMANN, LINDA J, PHD
PROFESSOR
UNIVERSITY OF WICONSIN
SCHOOL OF NURSING
MADISON, WI 53792

BENNETT, IAN MOORE, MD, PHD
ASSOCIATE PROFESSOR
DEPARTMENT OF FAMILY MEDICINE
AND COMMUNITY HEALTH
UNIVERSITY OF PENNSYLVANIA HEALTH SYSTEM
PHILADELPHIA, PA 19104

BOIVIN, MICHAEL J, MPH, PHD
PROFESSOR
DEPARTMENTS OF PSYCHIATRY AND
NEUROLOGY/OPHTHALMOLOGY
COLLEGE OF OSTEOPATHIC MEDICINE
MICHIGAN STATE UNIVERSITY
EAST LANSING, MI 48824

CUPERTINO, PAULA
ASSOCIATE PROFESSOR
DEPARTMENT OF PREVENTIVE MEDICINE
AND PUBLIC HEALTH
UNIVERSITY OF KANSAS MEDICAL CENTER
KANSAS CITY, KS 66160

DE ERAUSQUIN, GABRIEL A, MD, PHD
PROFESSOR AND DIRECTOR
PSYCHIATRY, NEUROLOGY AND NEUROSURGERY
ROSKAMP CHAIR OF BIOLOGICAL PSYCHIATRY
MORSANI COLLEGE OF MEDICINE
UNIVERSITY OF SOUTH FLORIDA
TAMPA, FL 33613

DECKELBAUM, RICHARD J, MD
PROFESSOR AND DIRECTOR
DEPARTMENT OF PEDIATRICS
INSTITUTE OF HUMAN NUTRITION
COLUMBIA UNIVERSITY
NEW YORK, NY 10032

DEPP, COLIN A, PHD
ASSOCIATE PROFESSOR
DEPARTMENT OF PSYCHIATRY
SCHOOL OF MEDICINE
UNIV. OF CALIFORNIA, SAN DIEGO
LA JOLLA, CA 92093

EHIRI, JOHN E, MPH, PHD
PROFESSOR AND DIRECTOR
DIVISION OF HEALTH PROMOTION SCIENCES
MEL AND ENID ZUCKERMAN COLLEGE OF PUBLIC HEALTH
UNIVERSITY OF ARIZONA
TUCSON, AZ 85724

EHRHARDT, STEPHAN , MPH, MD
ASSOCIATE PROFESSOR
DEPARTMENT OF EPIDEMIOLOGY
JOHNS HOPKINS SCHOOL OF PUBLIC HEALTH
BALTIMORE, MD 21205

ESSIEN, EKERE J, MPH, MD, DRPH
PROFESSOR
DEPARTMENT OF CLINICAL SCIENCES
AND ADMINISTRATION
COLLEGE OF PHARMACY
UNIVERSITY OF HOUSTON
HOUSTON, TX 77030

GANCE-CLEVELAND, BONNIE , PHD
LORETTA FORD PROFESSOR
DIVISION OF WOMEN, CHILDREN, AND FAMILY HEALTH
COLLEGE OF NURSING
ANSCHUTZ MEDICAL CAMPUS
UNIVERSITY OF COLORADO
AURORA, CO 80045

GIORDANI, BRUNO , PHD
PROFESSOR
DEPARTMENT OF PSYCHIATRY, NEUROLOGY, &
PSYCHOLOGY
SCHOOL OF NURSING
UNIVERSITY OF MICHIGAN
ANN ARBOR, MI 48109

HARRINGTON, DAVID P, PHD
PROFESSOR
DEPARTMENT OF BIOSTATISTICS AND STATISTICS
HARVARD UNIVERSITY & DANA-FARBER CANCER
INSTITUTE
BOSTON, MA 02115

HEFFRON, RENEE ANNETTE, MPH, PHD
DEPARTMENT OF GLOBAL HEALTH
THE UNIVERSITY OF WASHINGTON
SEATTLE, WA 98104

INTILLE, STEPHEN S, PHD
ASSOCIATE PROFESSOR
COLLEGE OF COMPUTER AND INFORMATION SCIENCE
AND BOUVE COLLEGE OF HEALTH SCIENCES
NORTHEASTERN UNIVERSITY
BOSTON, MA 02115

LELUTIU-WEINBERGER, CORINA , PHD
RESEARCH SCIENTIST
DEPARTMENT OF PSYCHOLOGY
HUNTER COLLEGE OF THE CITY
UNIVERSITY OF NEW YORK
NEW YORK, NY 10065

LEMMA, WULETA
ASSOCIATE PROFESSOR
DEPARTMENT OF GLOBAL COMMUNITY HEALTH
AND BEHAVIORAL SCIENCES
SCHOOL OF PUBLIC HEALTH AND TROPICAL MEDICINE
TULANE UNIVERSITY
NEW ORLEANS, LA 70112

LIU, LONGJIAN , MD, PHD
ASSOCIATE PROFESSOR
DEPARTMENT OF EPIDEMIOLOGY AND BIOSTATISTICS
DREXEL UNIVERSITY
PHILADELPHIA, PA 19102

LUQUE, JOHN S, MPH, PHD
ASSOCIATE PROFESSOR
DEPT. OF COMMUNITY HEALTH BEHAVIOR & EDUCATION
JIANN-PING HSU COLLEGE OF PUBLIC HEALTH
GEORGIA SOUTHERN UNIVERSITY
STATESBORO, GA 30458

MARIENFELD, CARLA B, MD
ASSISTANT PROFESSOR
DEPARTMENT OF PSYCHIATRY
YALE UNIVERSITY SCHOOL OF MEDICINE
NEW HAVEN, CT 06511

MURPHY, ROBERT L., MD
PROFESSOR AND DIRECTOR
CENTER FOR GLOBAL HEALTH
DEPARTMENT OF MEDICINE
FEINBERG SCHOOL OF MEDICINE
NORTHWESTERN UNIVERSITY
CHICAGO, IL 60611

MURRAY, MEGAN B, MPH, SCD, MD
PROFESSOR
DEPARTMENT OF EPIDEMIOLOGY
SCHOOL OF PUBLIC HEALTH
HARVARD UNIVERSITY
BOSTON, MA 02115

NACHEGA, JEAN B, MPH, MD, PHD
ASSOCIATE PROFESSOR
DEPARTMENT OF EPIDEMIOLOGY
SCHOOL OF PUBLIC HEALTH
UNIVERSITY OF PITTSBURGH
PITTSBURGH, PA 15261

POELLABAUER, CHRISTIAN , PHD
ASSOCIATE PROFESSOR
UNIVERSITY OF NOTRE DAME
NOTRE DAME, IN 46556

RAM, PAVANI K, MD
ASSOCIATE PROFESSOR
SCHOOL OF PUBLIC HEALTH
EPIDEMIOLOGY AND ENVIRONMENTAL HEALTH
DIRECTOR, OFFICE OF GLOBAL HEALTH INITIATIVES
STATE UNIVERSITY OF NEW YORK BUFFALO
BUFFALO, NY 14214

STCLAIRE, TAMARA
CHIEF INNOVATION OFFICER
XEROX COMMERCIAL HEALTHCARE
SACRAMENTO, CA 95837

STEINHUBL, STEVEN R, MD
DIRECTOR
DIGITAL MEDICINE
SCRIPPS TRANSLATIONAL SCIENCE INSTITUTE
LA JOLLA, CA 92037

THOMAS, JAMES C, MPH, PHD
ASSOCIATE PROFESSOR
DEPARTMENT OF EPIDEMIOLOGY
UNIVERSITY OF NORTH CAROLINA AT CHAPEL HILL
CHAPEL HILL, NC 275997400

TISCH, DANIEL J, MPH, PHD
ASSOCIATE PROFESSOR
SCHOOL OF MEDICINE
CASE WESTERN RESERVE UNIVERSITY
CLEVELAND, OH 44106

TORIOLA, ADETUNJI T, MPH, MD, PHD
ASSISTANT PROFESSOR
DEPARTMENT OF SURGERY
DIVISION OF PUBLIC HEALTH SERVICES
WASHINGTON UNIVERSITY SCHOOL OF MEDICINE
ST. LOUIS, MO 63110

WADE, ERIC , PHD
ASSISTANT PROFESSOR
DEPARTMENT OF MECHANICAL, AEROSPACE,
AND BIOMEDICAL ENGINEERING
UNIVERSITY OF TENNESSEE
KNOXVILLE, TN 37966

WALANI, SALIMAH R, MPH, MSN, PHD
DIRECTOR
GLOBAL HEALTH PROGRAMS
MARCH OF DIMES
WHITE PLAINS, NY 10605

WALSH, JULIA A, MD
ADJUNCT PROFESSOR
PUBLIC HEALTH
MATERNAL & CHILD HEALTH
UNIVERSITY OF CALIFORNIA BERKELEY
BERKELEY, CA 94720

XIAN, XIAOJUN , PHD
ASSOCIATE RESEARCH SCIENTIST
CENTER FOR BIOELECTRONICS AND BIOSENSORS
THE BIODESIGN INSTITUTE
ARIZONA STATE UNIVERSITY
TEMPE, AZ 85287

YOUNGBLOOD, G. MICHAEL
SENIOR AI SYSTEMS ARCHITECT AND RESEARCHER
INTERACTIVE INTELLIGENCE AREA
INTERACTION AND ANALYTICS LAB
PALO ALTO RESEARCH CENTER
PALO ALTO, CA 94304

MAIL REVIEWER(S)

AMAYA-BURNS, ALBA P, MD
ASSOCIATE PROFESSOR
DUKE KUNSHAN UNIVERSITY
JIANGSU, 215316
CHINA

FINKEL, MADELON , PHD
PROFESSOR
DEPARTMENT OF PUBLIC HEALTH
WEILL CORNELL MEDICAL COLLEGE
NEW YORK, NY 10065

KIENE, SUSAN MARIA, MPH, PHD
ASSOCIATE PROFESSOR
DIVISION OF EPIDEMIOLOGY AND BIOSTATISTICS
DEPARTMENT OF GLOBAL HEALTH
GRADUATE SCHOOL OF PUBLIC HEALTH
SAN DIEGO STATE UNIVERSITY
SAN DIEGO, CA 92182

KIRBY, BRIAN J, PHD
ASSOCIATE PROFESSOR
SIBLEY SCHOOL OF MECHANICAL & AEROSPACE ENG
COLLEGE OF ENGINEERING
KIRBY RESEARCH GROUP
CORNELL UNIVERSITY
ITHACA, NY 14853

MURUGESAN, MALLESH
PRESIDENT AND CEO
ABEYON
MIAMI, FL 33156

STELLING, JOHN , MPH, MD
CO-DIRECTOR
COLLABORATING CENTRE FOR SURVEILLANCE
OF ANTIMICROBIAL RESISTANCE
WORLD HEALTH ORGANIZATION
BOSTON, MA 02115

SCIENTIFIC REVIEW OFFICER

RICHON, ALLEN , PHD
SCIENTIFIC REVIEW OFFICER
CENTER FOR SCIENTIFIC REVIEW
NATIONAL INSTITUTES OF HEALTH
BETHESDA, MD 20892

EXTRAMURAL SUPPORT ASSISTANT

AKOMAH, STEPHEN
LEAD EXTRAMURAL SUPPORT ASSISTANT
CENTER FOR SCIENTIFIC REVIEW
NATIONAL INSTITUTES OF HEALTH
BETHESDA, MD 20892

Consultants are required to absent themselves from the room during the review of any application if their presence would constitute or appear to constitute a conflict of interest.
